# 3D‐Printed Organic–Ceramic Complex Hybrid Structures with High Silica Content

**DOI:** 10.1002/advs.201800061

**Published:** 2018-05-28

**Authors:** Efrat Shukrun, Ido Cooperstein, Shlomo Magdassi

**Affiliations:** ^1^ Casali Center for Applied Chemistry Institute of Chemistry The Center for Nanoscience and Nanotechnology The Hebrew University of Jerusalem Jerusalem 9190401 Israel

**Keywords:** 3D printing, digital light processing, hybrid organic–ceramic inks, sol‐gel, UV curable materials

## Abstract

Hybrid organic–inorganic sol gel inks that can undergo both condensation and radical polymerization are developed, enabling fabrication of complex objects by additive manufacturing technology, yielding 3D objects with superior properties. The 3D objects have very high silica content and are printed by digital light processing commercial printers. The printed lightweight objects are characterized by excellent mechanical strength compared to currently used high‐performance polymers (139 MPa), very high stability at elevated temperatures (heat deflection temperature >270 °C), high transparency (89%), and lack of cracks, with glossiness similar to silica glasses. The new inks fill the gap in additive manufacturing of objects composed of ceramics only and organic materials only, thus enabling harnessing the advantages of both worlds of materials.

3D printing is an additive manufacturing technology, which enables fabrication of complex structures and 3D objects, composed of a variety of materials. Recent interest in 3D printing has led to the development of novel ink compositions and printing technologies, such as for printing of polymer‐derived ceramics,^1^ hydrogels,^2^ smart objects composed of shape memory polymers,^3^ and electroactive polymers.^4^ Sol‐gel processes are well known as a simple route to obtain materials with unique properties. However, so far there have been no reports on sol‐gel based inks composition which can be 3D‐printed to fabricate 3D objects with high mechanical strength, thermal stability, and transparency, which have complex structures that cannot be fabricated by conventional methods.

All 3D printing techniques are based on sequential deposition of 2D patterns along the *Z*‐axis, in order to create a 3D structure, which corresponds to a computer‐aided designed (CAD) 3D model.^5^ The most affordable printing technology today, with the highest printing resolution, is stereolithographic 3D printing, in which 2D patterns are formed by selective light irradiation of photosensitive monomers, or oligomers in a liquid form, in presence of dissolved photoinitiators (PI). The PI is activated by the printer's light source that initiates the photopolymerization of the monomer/oligomer to form a 2D cured cross‐linked polymeric layer.^6^ Digital light processing (DLP) is a modified stereolithographic technology, which utilizes a digital micromirror device (DMD) to direct the UV light and to pattern the 2D image in one step. The DLP method enables printing of high‐resolution complex structures (1 µm layer thickness at the *Z*‐axis and 20 µm at *XY*‐axes) composed of various materials, without the need of support materials, and at a high processing speed.^2^

For the radical polymerization initiated by a UV light source at the DLP printer, there is a need for organic photopolymerizable groups. Therefore, most of the structures manufactured by stereolithography are mainly composed of organic polymers with a large variety of optical properties, (transparency or colored), mechanical properties, (flexibility or hardness), and biocompatibility. However, due to the organic backbone, such materials suffer from the lack of toughness and low thermal stability.

A combination of organic and inorganic components is a perspective route for obtaining materials with improved properties. For example, Kotz et al. have reported on inks containing silica nanoparticles dispersed in organic photopolymerizable resin,^7^ which after printing, the 3D object decomposes at high temperature and results in a ceramic object. A different approach is based on using hybrid molecules that have UV‐polymerizable moiety and a group that can undergo a sol‐gel process to yield hybrid objects. These objects contain properties which benefit the organic and inorganic components such as high mechanical strength, optical transparency, and high thermal and chemical resistance. The properties of such hybrid materials can be tailored by changing the type and ratio between the inorganic and the organic components,^8^ thus making them suitable for various applications. They can be used in the optics industry as waveguides or electro‐optical devices,^9^ address the need for solvent and heat‐resistant microfluidic reactors,^10^ and for applications such as solar cells,^11^ dental filling,^11^ and art.

The use of hybrid UV‐cured materials in photolithography has been already reported in the literature.^12^ The precursors for producing these materials are usually based on organically modified silanes^13^ such as epoxy‐based 3‐glicydyloxypropyl‐trimethoxysilane (GPTMS), acrylic‐based 3‐methacryloxypropyltrimethoxysilane (MAPTMS), acryloxypropyltrimethoxysilane (APTMS), and methacrylphenyl polyhedral oligomeric silsesquioxanes (MA‐POSS).^14^ However, these organic–inorganic hybrids usually have a high content of organic material. Therefore, their material properties, for example, low thermal stability and low mechanical strength of the final product, are more characteristic for organic polymers than for inorganic material.[[qv: 8b]] Hybrid UV‐active preceramic polymers, such as a mixture of (mercaptopropyl) methylsiloxane, vinylmethoxysiloxane,[[qv: 1a]] and methyl‐silsesquioxane preceramic polymer[[qv: 1b]] as inks for stereolithography printing, was used to achieve ceramics objects, but they lacked transparency. We propose to overcome these limitations by designing new hybrid organic–inorganic sol‐gel‐based inks with very high silica content, by using materials that will narrow the gap between ceramic and organic 3D objects. The sol‐gel process is a well‐known approach for the production of inorganic materials at low temperatures.^15^ Combining a small amount of modified metal‐alkoxy precursors with conventional metal‐alkoxy sol‐gel precursors will enable the formation of oligomer sol with high silica content that can be both polymerized under UV‐light and undergo a sol‐gel process. Synthesis of such photopolymerizable sol with high silica content was demonstrated for 3D structures that were prepared within a mold.[[qv: 15c]] A fast sol‐gel technique was used to create a glassy‐like material, which resulted in a 3D structure with minimum shrinkage and no cracks. However, due to the high viscosity of the obtained sol, it was not printable. Another report describes 3D printing by DLP of polyethylene glycol diacrylate (PEGDA) mixed with sol‐gel precursors, followed by postprinting exposure to acid vapors to enable the hydrolysis and the condensation of the sol‐gel precursors,^16^ which resulted in increased hardness of the matrix. However, due to the high organic content (≈89%) and the composition of the starting material, the printed structures had low mechanical strength (32 MPa).

Here, we report on new, 3D printable sol‐gel‐based ink compositions, which will enable fabrication of 3D objects, with unique properties, by the DLP additive manufacturing technology. The liquid ink is composed of hybrid UV‐curable metal‐alkoxy oligomers that can undergo both a sol‐gel process and radical polymerization. The synthesis of hybrid photopolymerizable oligomers is performed by a sol‐gel process, and the curing of the 2D layers of the 3D structures is done via photopolymerization during printing. After printing, during aging, a polycondensation process occurs, in which condensation reactions take place between adjacent silanol groups, forming a stronger network.^17^ This approach enables fabrication of 3D objects with extremely high silica content. They are transparent and crack free, have very high mechanical strength, are stable at elevated temperatures, and possess glossiness similar to silica glass.^18^

More specifically, the hybrid ink was prepared via the sol‐gel process by combining a silicon alkoxy precursor with one photopolymerizable acrylate group, 3‐acryloxypropyltrimethoxysilane (APTMS) with conventional sol‐gel monomers, tetramethyl orthosilicate (TMOS), and methyl trimethoxysilane (MTMS), under acidic conditions (**Figure** **1**a,b). The inks also contained a dissolved photoinitiator, 2,4,6‐trimethylbenzoyldiphenylphosphineoxide (TPO).

**Figure 1:**
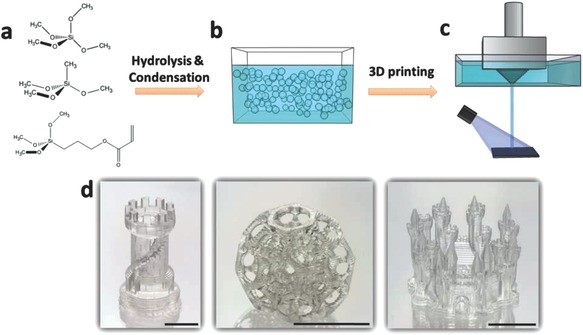
Scheme of the fabrication process: a) three silica alkoxides used for synthesis. b) UV‐curable hybrid oligomers made by sol‐gel process; c) selective photopolymerization of the hybrid oligomers (gelation) by the DLP printing technique. d) Typical aged 3D‐printed structures (scale bar, 1 cm).

To print the sol‐gel‐based ink by the DLP technology, the ink needs to remain in its liquid form and, therefore, it is crucial to increase its gelation time. This can be achieved by keeping a low pH (≈3) during the synthesis, near the isoelectric point of the silica sol,^19^ and by further evaporation of the water.[[qv: 15b]] After reaching a stage in which the composition can be rapidly cured by UV light (by testing a small sample), it is cooled down to room temperature, and the reaction vessel is closed in order to slow down the condensation reaction. The ink viscosity was 5.29 ± 0.02 cP (Figure S1, Supporting Information) at the end of the ink synthesis process. The printing process could be performed with inks that were stored at room temperature for at least one week after preparation. After a longer storage time, although the printing could be performed, the aging process resulted in printed objects with cracks. It should be noted that there are several approaches to extend shelf life for several months, such as by adding solvents and evaporating them prior to formation of the object.^20^

The printing of the hybrid oligomers was performed by a commercial DLP printer, in which the UV‐curing light was radiated from the bottom of the reservoir to the monomers bath, thus resulting in objects which adhere to the printing platform (Figure 1c). The *X*–*Y* axes resolution of the printer used in this study was 39 µm, and the used *Z*‐axis resolution was in the range of 50–350 µm (the minimal layer thickness was 1 µm) (**Figure** **2**). The 3D digital models were designed by commercial computer‐aided design software (CAD) or downloaded from open source CAD websites. The files were adapted and sliced by the printer's software according to the desirable *Z*‐axis resolution.

**Figure 2:**
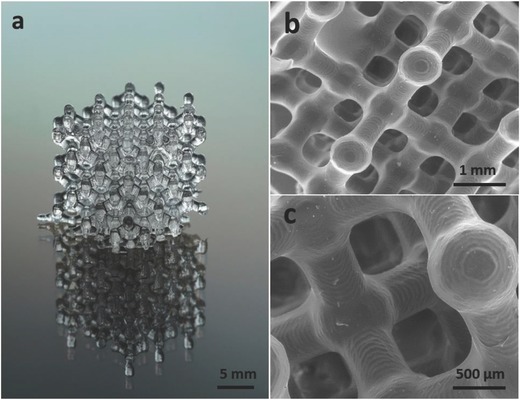
a) Printed structure with 50 µm resolution at the *Z*‐axis aged at 60 °C for 5 d. b,c) SEM images of the same structure.

During printing, localized radical photopolymerization occurs, forming a covalent bond between the acrylic groups in the oligomers, thus enabling initial fixation of the desired structure. The resulting printed structure at this stage is not rigid, and is a gel‐like material. Following the initial formation of the object, it is left for an aging process. During this process a sol‐gel polycondensation reaction occurs, which finally results in a hybrid, ceramic–organic transparent 3D object with a complex structure and superior properties (Figure 1d).

We have evaluated two types of aging processes: (a) aging at room temperature for the duration of up to one month, (b) aging for 7 d at RT, followed by 3 d at 60 °C and then at higher temperatures, ranging from 60 to 400 °C for 2 h. As shown in **Figure** **3**a, the amount of silica in the 1 h aged sample is 65.8 ± 0.5 wt%, as determined by weight loss after heating to 1000 °C, a temperature in which there are no organic material residues (absence of carbon was confirmed by energy‐dispersive spectroscopy (EDX) and elemental analyses). As the aging time increases, the amount of silica increases, reaching a maximum of 80 ± 2 wt% after 60 d at room temperature. This remains constant even after 489 d, as presented in the inset of Figure 3a. As seen in the thermogravimetric analysis (TGA) curves, there are three main regions of mass loss: (a) from room temperature to 150 °C, mass loss is due to the evaporation of free water that is present as a solvent and reaction by‐product, methanol being a reaction by‐product, (b) at 150–400 °C, mass loss is due to the evaporation of chemisorbed water and the condensation reaction,^21^ and (c) the initial decomposition of organic residues. The mass loss in the range of 400–700 °C is a result of decomposition of the organic residues. Figure 3b presents the TGA curves for the 3D‐printed structures which were aged for 7 d at RT, 3 d at 60 °C, and 2 h at higher temperatures, up to 400 °C. These samples show no weight loss while heated to 150 °C, since all unbound water and methanol were already evaporated during the aging stage. After heating at 250 °C for 2 h, the fraction of silica in the object was 84 ± 1%, and after 2 h at 400 °C the amount of silica in the object was as high as 90 ± 1 wt%. It should be noted that the structures of the printed objects were retained after the two aging processes for all samples, even at high temperatures. Therefore, we measured the heat deflection temperature (HDT), which is an important indicator for high‐performance polymeric materials. The HDT of a sample that was aged for 7 d at room temperature, followed by 3 d at 60 °C and 2 h at 250 °C, was found to be above 270 °C, which is much higher compared to current commercial 3D inks (Formlabs High Temp,^22^ Stratasys High Temperature,^23^ 3D SYSTEMS Accura Bluestone,^24^ DSM Somos NanoTool,^25^ and Carbon CE221^26^) as can be seen in Figure 3c. To demonstrate this excellent property, we printed objects using our ink (at the same aging process), and high‐temperature commercial inks (Stratasys High Temperature, and Asiga fusionGRAY^27^), and tested their thermal stability by placing 50 g weight and heating to 270 °C for 2 h. The results showed that the objects printed using the hybrid ink had no deflection, compared with the best commercial materials (Figure 3d).

**Figure 3:**
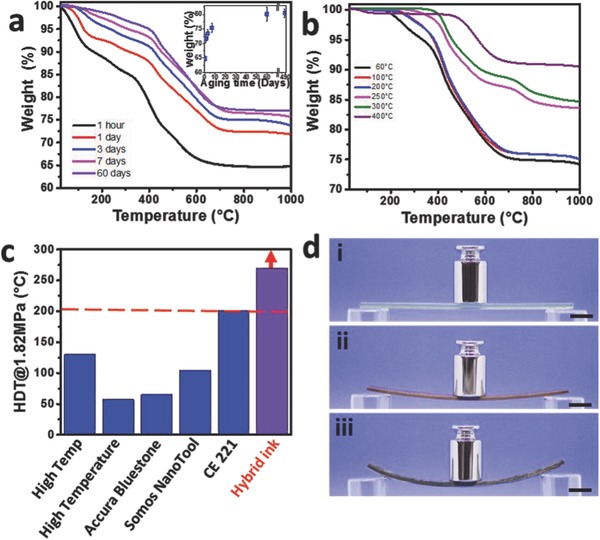
a) TGA in air of printed samples after aging at RT as a function of aging duration. Inset shows weight of the samples at longer aging time. b) TGA in air of printed samples after aging all samples for 7 d at RT, followed by 3 d at 60 °C and storage for 2 h at higher temperatures. c) HDT comparisons of inks for high thermal resistance materials. d) Image of samples printed with the hybrid ink (i), Stratasys high temperature (ii), and Asiga fusionGRAY (iii), after heating at 270 °C for 2 h while placing 50 g weight.

The mechanical strength of 3D‐printed objects depends on the properties of the constituent material, its porosity and the architecture of the structure itself.[[qv: 1a]] In sol‐gel processes, the pH during the reaction enables control of the properties of the formed silica gel.^28^ In general, acid‐catalyzed hydrolysis and condensation tend to form weakly branched linear molecules,[[qv: 15b,29]] which form microporous dense structures that enhance their mechanical strength. Furthermore, the polycondensation reaction throughout the aging process[[qv: 15b]] leads to formation of siloxane bonds that contribute to the formation of a stronger matrix,[[qv: 15c,30]] and eventually control the high mechanical strength of the 3D structure. The condensation process also results in the shrinkage of the object and the increase of its density.

**Figure** **4** presents the shrinkage (a), density (b), and maximum compressive stress (c) as a function of the aging process for printed objects. The aging process, for all samples, was 7 d at RT followed by 3 d at 60 °C, and then each sample was aged for 2 h at temperatures ranging from 60 to 400 °C. It was found that after 7 d of aging at RT only, the shrinking reached a plateau value of 32.4% (inset in Figure 4a) without the formation of cracks, and the density of the printed structure (Figure 4b) was 1.330 ± 0.001 g cm^−3^ (1.364 ± 0.006 g cm^−3^ after 3 d aging at 60 °C). As the aging temperature was increased (up to 200 °C), polycondensation and further solvent evaporation from the structure continued, causing a further increase in shrinkage and mass loss, which resulted in a density decrease. As the temperature increased from 200 to 300 °C, the density, the shrinkage and the mass (Figure 3a) remained nearly constant. At 400 °C the shrinkage increased and the density decreased due to the weight change of the samples caused by the decomposition of the organic residues. It is important to note that the shrinkage is isotropic (as found by length measurements in *x*,*y*,*z*‐axes of a printed lattice object, Figure S2, Supporting Information), hence, it did not result in any deformation of the 3D structure.

**Figure 4:**
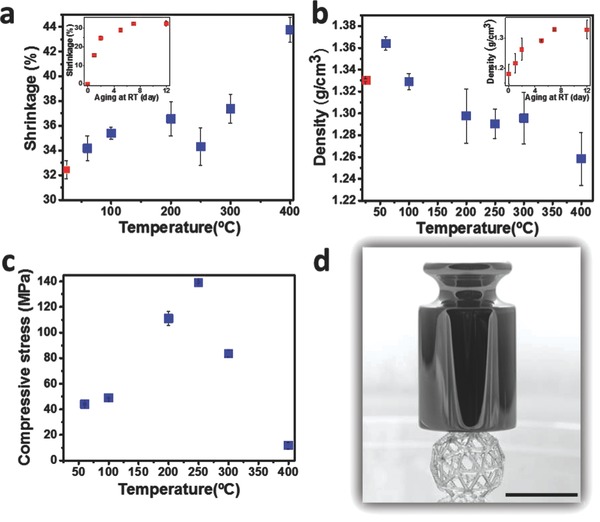
a) Shrinkage, b) density, and c) maximum compressive stress as functions of aging temperature for printed samples aged for 7 d at RT, 3 d at 60 °C, and for 2 h at higher temperatures (heating rate of 1 °C min^−1^). Insets show shrinkage (a) and density (b) as functions of aging time at RT. d) Printed 3D objects after aging at 250 °C for 2 h while placing 50 g weight on the sample (scale bar, 1 cm).

To evaluate the mechanical strength of the printed structures, rectangular objects were printed, and the maximum compression stress was measured for samples that were aged at the same process as above. As shown in Figure 4c, an increase in final aging temperature led to improved mechanical strength, reaching 139.1 ± 0.7 MPa at 250 °C. As can be seen from Figure S3b (Supporting Information), compared to other materials printed by SLA 3D printing technologies, the hybrid material had the highest mechanical strength. Compared to industrial high‐performance polymers, the mechanical strength was almost the same as ULTEM^31^ polymer (polyetherimide) and better than PEEK^32^ (polyether ether ketone) (Figure S3b, Supporting Information). We also measured the flexural strength of samples that were aged for 7 d at RT, followed by 3 d at 60 °C and then aged for 2 h at 250 °C. We found that the material had a flexural strength of 1.31 ± 0.05 MPa. It should be noted that elongation at break test could not be performed due to breaking of the samples by the instrument clamps. The internal morphology of the printed objects was evaluated by scanning electron microscopy (SEM) imaging of cross sections of as‐printed and aged samples. As shown in Figure S4 (Supporting Information), the as‐printed object had a slightly porous structure, while the aged sample was very smooth and without any observable pores. Figure 4d illustrates the high mechanical strength of a printed hollow ball with only 0.6 mm thick lines that does not collapse under a weight of 50 g. At 300 °C, the mechanical strength decreases significantly, and this is attributed to the formation of cracks within the printed structure. Furthermore, we investigated the mechanical strength of the printed objects which were aged at 60 °C for various time durations. As seen in Figure S5 (Supporting Information), the compressive strength of the objects became larger with aging time, and the best result was obtained after 26 d of aging, and compressive stress of 69 ± 3 MPa.

To evaluate the transparency of the printed 3D objects, cubes with 5 × 5 × 5 mm^3^ dimensions were printed, and aged as above. **Figure** **5**a,c presents the transparency of the samples. As seen in the visible range (400–760 nm) the transmittance is 87–89% at wavelengths higher than 430 nm. The low transmittance at lower wavelengths is due to the high absorbance of the PI at this wavelength (Figure S6, Supporting Information). As the temperature increases to 300 and 400 °C, the object becomes yellowish and the transmittance decreases (transmittance of 83% for samples heated at 300 °C, and only 25% for samples heated at 400 °C). This is due to the formation of cracks and the partial degradation of the organic residues, as shown in Figure 5b. For most of the solvents that were tested, the hybrid objects have also shown very high solvent resistivity (Table S1, Supporting Information).

**Figure 5:**
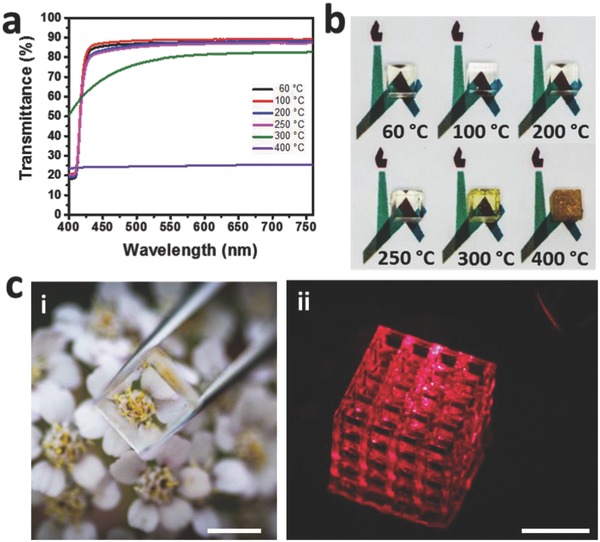
a) Transmittance of printed 3D objects. b) Objects after heat treatment at various temperatures, for 2 h at a heating rate of 1 °C min^−1^. c) (i) Printed 3D objects after aging at 250 °C (heating rate of 1 °C min^−1^) (scale bar, 5 mm). (ii) Laser light scattering of printed object (scale bar, 1 cm).

In conclusion, a new hybrid, organic–inorganic sol‐gel ink that can undergo both condensation and radical polymerization was developed. This composition enables fabrication of 3D objects with very high silica content by DLP technology. The printed objects are characterized by excellent mechanical strength compared to currently used high‐performance polymers (139 MPa). They are also characterized by very high stability at elevated temperatures (HDT > 270 °C), high transparency (89%), and lack of cracks, with glossiness similar to that of silica glass. The formed objects have a density only slightly above that of common plastics. Such inks pave the way for the printing of various complex and lightweight structures and objects which can be used in additive manufacturing processes while using commercially available low‐cost printers for the production of microfluidic reactors, and molds for the plastics and dental industry. These new inks will narrow the gap in additive manufacturing of objects composed of ceramics only and of organic materials only, thus enabling harnessing the advantages of both worlds of materials.

## Experimental Section

*Ink Composition*: The ink was prepared as follows: First, the monomers TMOS (tetramethylorthosilicate, 98%, Sigma Aldrich), MTMS (trimetoxymethylsilane, 98%, Sigma Aldrich), and APTMS (3‐acryloxypropyltrimethoxysilane, Gelest, USA) were mixed at a weight ratio of 3:15:2. Then 16 wt% of 0.5 × 10^−3^ m of HCl solution (Sigma Aldrich) and 1 wt% of TPO (2,4,6‐trimethylbenzoyl‐diphenyl‐phosphineoxide, BASF, Germany) were added to the monomers' mixture. The composition that was achieved was sealed in a dark vessel and stirred at 50 °C for 30 min, after which the temperature was increased to 70 °C and the vessel was opened. The composition was kept under stirring for additional 120 min, until the formed oligomers could form a gel matrix upon exposure to UV light (test preformed in a mold). Then the reaction vessel was closed and cooled down to room temperature.

*3D Printing*: A predesigned model was 3D‐printed using a DLP 3D printer (Freeform PICO 2, Asiga, Australia). This printer operates by UV‐LED light source (405 nm), with light intensity of 27 mW cm^−2^. The printer monomer bath was filled with the hybrid ink, and the exposure time which was suitable for obtaining the 3D objects was between 2 s for layer thickness of 50 µm and 5 s for layer thickness of 350 µm. After 3D printing, the structures were kept in a sealed vessel at room temperature for 7 d for further gelation, then, in open vessel, at 60 °C for 3 d to continue the aging process.

*Characterization*: TGA of printed samples was performed with a TGA/DSC1 stare system Mettler–Toledo in the range of 25–1000 °C at a heating rate of 1 °C min^−1^.

Mechanical tests were performed with Instron 4502 Universal Testing Machine. The measurements of the compression strength were done on printed and aged rectangular models with dimensions of 1 × 1 × 2 cm^3^ with *Z*‐axis resolution of 350 µm. The three‐point bend test was done on printed and aged models with dimensions of 127 mm × 12.7 mm × 3.2 mm that was cured in a mold.

Shrinkage was measured by a caliber for printed and aged cube models with a size of 5 × 5 × 5 mm^3^.

The light microscope images of lattice shaped structure (with printed layer thickness of 175 µm, and aging time of six months at room temperature) were taken by CX41microscope (Olimpus).

SEM images of 1 × 1 × 1 cm^3^ diamond‐like printed structure were taken by high‐resolution scanning electron microscope (HRSEM) Sirion (FEI company, USA).

EDX of the samples after 1000 °C was performed using Environmental SEM Quanta 200 (FEI company, USA) equipped with an EDX spectroscopy probe (Oxford X‐MAX, Oxford Instruments).

The determination of carbon was performed using the Thermo Scientific FLASH 2000 HT Elemental Analyzer (Thermo Scientific).

The transmittance measurements of a printed cube with dimensions of 5 × 5 × 5 mm^3^ and *z*‐axis resolution of 350 µm were performed with a UV spectrophotometer (UV‐1800, Shimadzu).

HDT measurement was performed according to ASTM D648 standard with load of 1820 kPa and heating speed of 120 °C (HDT/VICAT plus, Davenport, AMETEK). Note: the instrument reached its maximal temperature limit (270 °C) for all the duplicates, hence the exact HDT of the material remains unknown. The samples that demonstrated the HDT were cured by UV light in a mold. The dimensions of the samples were 127 mm × 12.7 mm × 3.2 mm (after the aging of the hybrid material), and their thermal stability was tested by placing 50 g weight while heating at 1 °C min^−1^ to 270 °C for 2 h, under atmospheric environment.

The viscosity of the ink was measured using a HAAKE RheoStress 6000 (Thermo Scientific) with a C60/1° Ti polished cone, and at shear rates between 0.1 and 100 s^−1^ at 25 °C. The ink showed Newtonian fluid behavior.

## Conflict of Interest

The authors declare no conflict of interest.

## Supporting information

Supplementary
