# Supplementary material for: 3D‐Printed Organic–Ceramic Complex Hybrid Structures with High Silica Content
Source: Adv Sci (Weinh). 2018 May 28;5(8):1800061. doi: 10.1002/advs.201800061 (PMC6096996; doi:10.1002/advs.201800061)
Supplement: Supplementary file 1 — Supplementary [file ADVS-5-1800061-s001.pdf]

Copyright WILEY-VCH Verlag GmbH & Co. KGaA, 69469 Weinheim, Germany, 2018.

ADVANCED SCIENCE Open Access

Supporting Information

for *Adv. Sci.,* DOI: 10.1002/advs.201800061

3D-Printed Organic–Ceramic Complex Hybrid Structures with High Silica Content

*Efrat Shukrun, Ido Cooperstein, and Shlomo Magdassi**

Copyright WILEY-VCH Verlag GmbH & Co. KGaA, 69469 Weinheim, Germany, 2017.

Supporting Information

3D printed organic-ceramic complex hybrid structures with high silica content

*Efrat Shukrun, Ido Cooperstein, and Shlomo Magdassi**

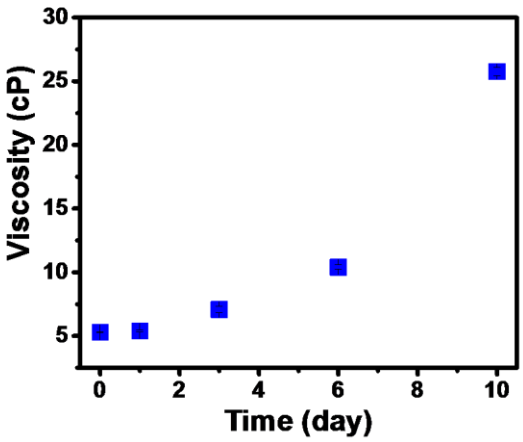

Figure S1: Viscosity measurements of the hybrid ink.

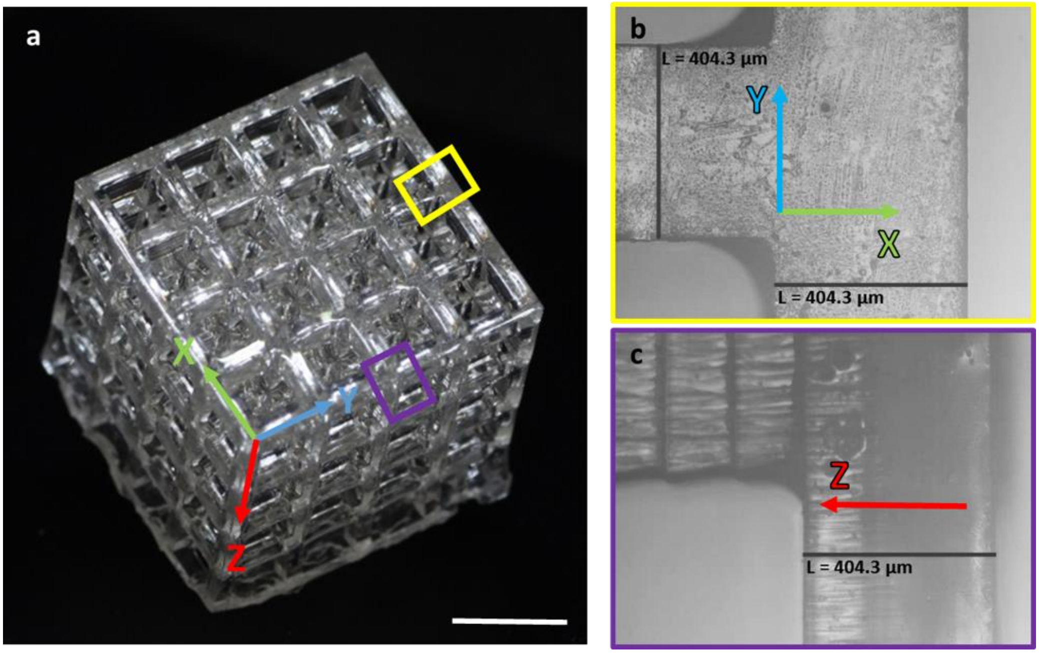

Figure S2: a) Image of symmetric lattice shaped printed structure (printed by printer settings of 150µm layer thickness) aged for six months at room temperature, the shrinkage in each axis of this object was about 13% (scale bar 5mm). b-c) Light microscope images showing the length of X, Y and Z axis of the aged structure, having exactly the same length presenting the isotropic shrinkage of the object.

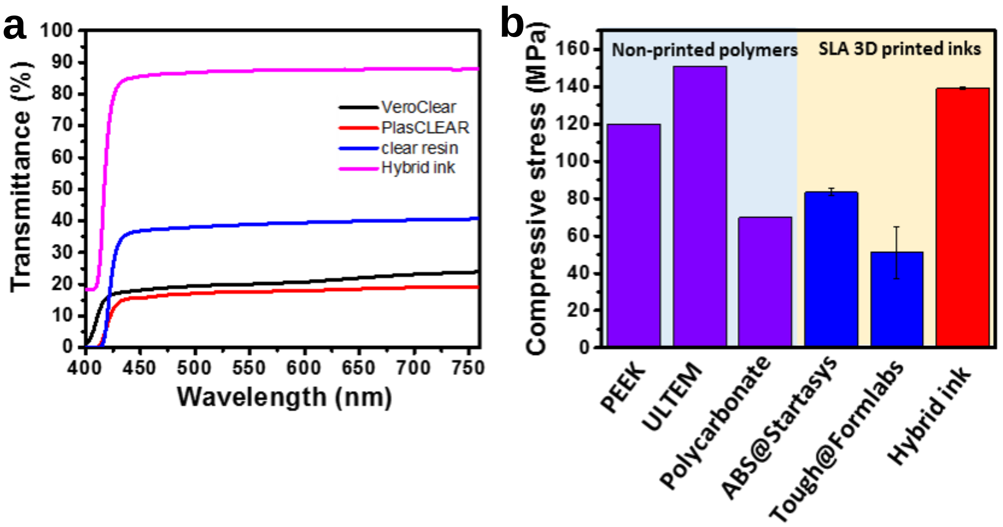

Figure S3: a) Transparency of the printed samples at a size of 20mm*20mm*2mm, with Z-axis resolution of 350µm (except of the VeroClear with Z-axis resolution of 25µm), at their companies printers. VeroClear at the Object30 (Stratasys), PlasCLEAR at Freeform PICO2 (Asiga) and clear resin at Formlabs +1(formlabs). The transparency was measured after printing, with no additional treatments from the Z-axis of the samples. d) Comparison of compressive strength between non-printed polymers, commercially available UV curable inks for SLA and our hybrid ink.

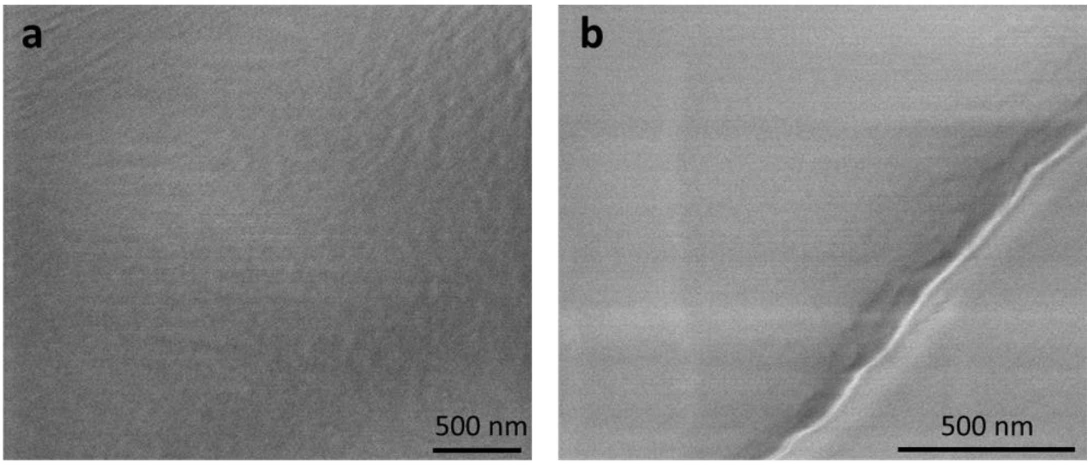

Figure S4: SEM images of cross-sections of (a) as-printed sample, (b) 47 days aged sample at room temperature.

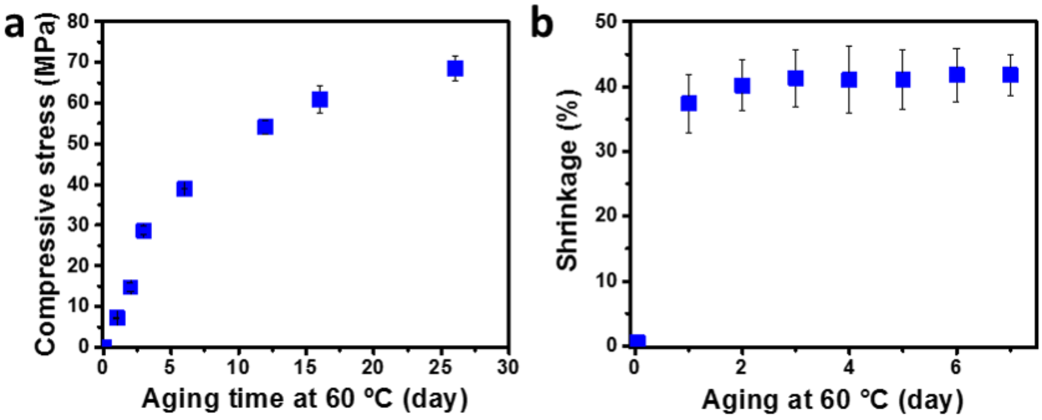

Figure S5: Measurements of printed samples aged at 60°C. a) Maximum compressive stress as a function of aging duration at 60°C, b) shrinkage measurements.

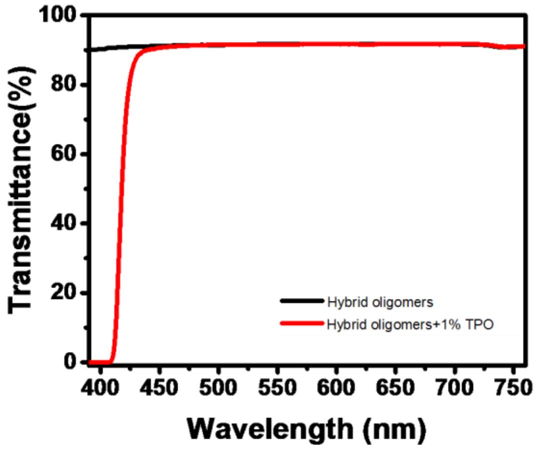

Figure S6: Transparency of the hybrid oligomers with and without TPO.

Solvent	solvent resistance
Ethylene Glycol	✓
Water	✓
Isopropanol	✓
Chloroform	✓
Acetone	The sample disintegrated
DMSO	The surface of the sample etched

Table S1: Solvents resistance of the printed hybrid samples after 48h.
